# Composite dietary antioxidant index is associated with the prevalence of metabolic syndrome in females: results from NHANES 2011–2016

**DOI:** 10.3389/fnut.2025.1529332

**Published:** 2025-03-27

**Authors:** Weili Liu, Yingying Xu, Liling Xiao, Ke Li, Qiang Liu

**Affiliations:** ^1^Shifang People's Hospital, Shifang, China; ^2^School of Life Sciences and Technology, University of Electronic Science and Technology of China, Chengdu, China

**Keywords:** NHANES, metabolic syndrome, CDAI, estrogen, females

## Abstract

**Objective:**

This study sought to investigate the association between metabolic syndrome (MetS) and Composite Dietary Antioxidant Index (CDAI) in females, with the goal of informing evidence-based prevention and clinical management strategies for MetS.

**Methods:**

The 2011–2016 National Health and Nutrition Examination Survey (NHANES) recruited a total of 2,790 female participants and screened 1,562 participants for estrogen non-deficiency. The diagnosis of MetS was based on criteria set by the National Cholesterol Education Program-Adult Treatment Panel III. The CDAI was calculated according to the intake of 10 dietary antioxidants. Multivariable logistic regression was performed to investigate the relationship between the CDAI and MetS in females. We also performed restricted cubic splines, two-piecewise linear regression, and subgroup analysis in further analysis.

**Results:**

Our analyses demonstrated a significant inverse association between the Composite Dietary Antioxidant Index (CDAI) and metabolic syndrome (MetS) prevalence in females. Restricted cubic spline analysis indicated a linear dose–response relationship (*p* for linearity = 0.029), with two-piecewise linear regression analysis revealed an inflection point at 1.99. Below 1.99, each unit increase in the CDAI was associated with a 2% reduction in the risk of MetS in females; above 1.99, the risk reduction was 1%. Participants without MetS were significantly younger than those with MetS (43.49 ± 16.04 vs. 54.77 ± 15.52 years, *p* < 0.001). Notably, estrogen levels also were negatively correlated with the prevalence of MetS. Subgroup analysis revealed that the relationship between the CDAI and MetS remained consistent across all subgroups.

**Conclusion:**

In the female population, CDAI levels exhibited an inverse relationship with the prevalence of metabolic syndrome, and estrogen levels demonstrated a negative correlation with its incidence.

## 1 Introduction

Metabolic syndrome (MetS) is a condition characterized by a clustering of metabolic risk factors. It is defined by the World Health Organization as a pathological condition characterized by abdominal obesity, insulin resistance, hypertension, and hyperlipidemia (1). Given its high prevalence and severe consequences, MetS has become a global problem, placing a huge economic burden on societies and health systems in the future (2–5).

However, women with MetS bear a greater health burden. Studies show that females have a higher prevalence of MetS than men (5), and female patients tend to experience more severe symptoms than males (6, 7). Previous research suggests that MetS in middle-aged females may contribute to or exacerbate pain, sleep disturbances, sexual dysfunction, and mood alterations, likely due to aging and chronic inflammation (8). A systematic review and meta-analysis further revealed that MetS in females is associated with an increased risk of several malignancies, including endometrial, pancreatic, and breast cancers. The strongest associations were seen in sex-specific cancers like endometrial cancer and postmenopausal breast cancer (6). Remarkably, postmenopausal females with MetS have a twofold higher risk of developing breast cancer than those without MetS (8).

Evidence indicates that females with MetS experience more severe symptoms than men, possibly due to sex-related hormonal factors. Estrogen plays a crucial role in modulating inflammation and metabolic homeostasis in females (9, 10). It regulates insulin resistance (11), energy metabolism (12), and lipid metabolism (13, 14). The hormone exhibits dual functionality in oxidative stress regulation, providing antioxidant protection through receptor-mediated mechanisms (15), while also orchestrating cellular defense systems against oxidative damage (16). Pathologically, the chronic inflammation and oxidative stress characteristic of MetS are well-established contributors to carcinogenesis (8, 15). Recent studies suggest that ERα-mediated mitochondrial energy regulation represents a key pathway underlying estrogen’s metabolic protective effects (16). However, the postmenopausal decline in estrogen levels promotes macrophage infiltration in adipose tissue, worsening insulin resistance via TNF-α/IL-6-JNK pathway activation (17).

Estrogen fluctuations, mediated through cyclic variations in nuclear receptor ERRα-regulated mitochondrial biogenesis (18), constitute an inherent physiological characteristic in females. Targeting the menopausal decline in estrogen through dual strategies—suppressing NADPH oxidase activity to mitigate oxidative stress while enhancing endogenous antioxidant defenses (e.g., superoxide dismutase (SOD) and catalase)—may effectively reduce the risk of MetS (19). Therefore, antioxidant protection may yield significant benefits for females with MetS, offering greater preventive value than treatment alone. Mechanistically, oxidative stress drives MetS pathogenesis via NF-κB-mediated inflammation (20), insulin signaling disruption, and adipocyte dysfunction (21). Several studies have confirmed the role of dietary antioxidants in counteracting oxidative stress (22–25). For instance, serum levels of carotenoids, particularly α and β-carotene, as well as retinyl esters, exhibit an inversely association with MetS (26). Additionally, abnormal vitamin A metabolism contributes to damage and plays a key role in MetS in a gut microbiota-dependent manner (27) while dietary vitamin E levels are inversely correlated with MetS (28, 29).

Therefore, mitigating oxidative stress may represent a viable preventive and therapeutic approach for managing metabolic syndrome (MetS) in female populations. Notably, while previous investigations predominantly focused on isolated antioxidant components, emerging evidence underscores the critical role of holistic dietary patterns in modulating oxidative-inflammatory pathways (30). The Comprehensive Dietary Antioxidant Index (CDAI), a validated metric reflecting synergistic antioxidant capacity, was developed based on its cumulative inhibitory effects on pro-inflammatory mediators, including tumor necrosis factor-alpha (TNF-α) and interleukin-1 beta (IL-1β). Its integrates quantitative assessments of multiple dietary antioxidants—vitamins A, C, and E, alongside manganese (Mn), selenium (Se), and zinc (Zn), etc., thereby providing a comprehensive characterization of individual antioxidant profiles (31), and has been applied in several studies (32–34).

Despite emerging evidence suggesting a non-linear association between MetS and the CDAI, critical knowledge gaps remain (35). Existing studies only found sex-specific differences in association between MetS and CDAI (36), and the stratified regression analyses indicate CDAI-MetS associations may be unique to female populations. However, the biological mechanisms underlying this sexual dimorphism, particularly the potential mediating role of estrogen in modulating antioxidant-metabolic interactions, remain inadequately explored. Since antioxidant supplements have shown no effect on MetS prevention, this study focused on dietary antioxidants (excluding supplements) to better reflect real-world nutritional exposures. In summary, this study aimed to quantify the CDAI-MetS association in females and investigate the role of estrogen in this relationship.

## 2 Materials and methods

### 2.1 Data source

**Figure 1:**
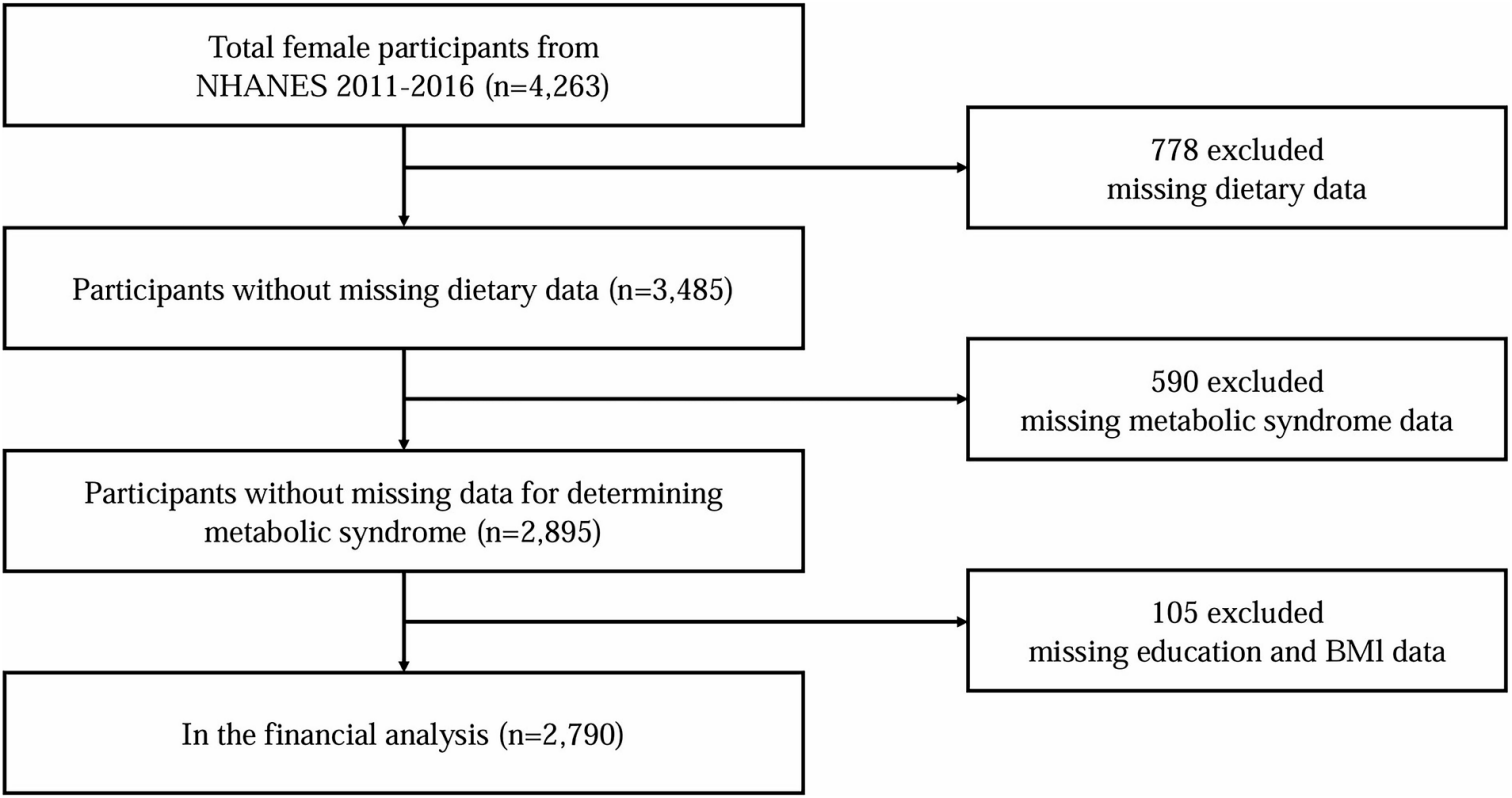
Flowchart of study population selection, NHANES 2011–2016.

All participant information and relevant data for this study were obtained from the National Health and Nutrition Examination Survey (NHANES) database, which is conducted by the National Center for Health Statistics, a division of the Centers for Disease Control and Prevention. For this study, we downloaded consecutive datasets from 2011 to 2016 (Figure 1), which were analyzed to accurately assess the relationship between a healthy CDAI index and MetS.

### 2.2 Exposure and outcomes

The NHANES database collects participants’ food intake over two consecutive days using 24-h dietary recall interviews. The first interview was conducted face to face, and the second was done over the phone 3–10 days later. The CDAI for all participants was calculated using the method recommended by Wright (Equation 1), incorporating 10 vitamins and minerals from food sources (vitamins A, C, E, selenium, zinc, alpha-carotene, beta-carotene, lycopene, lutein, and zeaxanthin, with lutein and zeaxanthin counting as a single metric). The intake of each antioxidant was standardized by subtracting the mean intake and then dividing by the standard deviation (where *x* represents the intake of individual dietary antioxidants and $\bar{x}$ represents the average intake of each component; SD is the standard deviation of the mean).


(1) $$\mathrm{CDAI}=\sum_{n=1}^{9}\frac{x-\bar{x}}{SD}\text{.}$$

In this study, we used the National Cholesterol Education Program-Adult Treatment Panel III (NCEP-ATP III) for the diagnosis of MetS. The NCEP-ATP III criteria are based on five measures: abdominal obesity, elevated triglycerides, lowered high-density lipoprotein (HDL) cholesterol, elevated blood pressure, and elevated fasting blood glucose. MetS is diagnosed when three or more of the following five criteria are met: (1) waist circumference ≥102 cm in men or ≥88 cm in females; (2) serum triglyceride ≥150 mg/dL; (3) serum HDL cholesterol <40 mg/dL in men or <50 mg/dL in females; (4) fasting blood glucose ≥100 mg/dL or use of hypoglycemic drugs; (5) blood pressure ≥130/85 mmHg or receiving relevant medication.

### 2.3 Covariates

To assess the impact of potential confounders, several important covariates were selected, including age, race and ethnicity, education level, marital status, drinking, smoking, body mass index (BMI), diabetes, and hypertension. Categorical variables included race and ethnicity (Non-Hispanic White people, Non-Hispanic Black people, Mexican American, and Other), education level (High school, Above high school, and Other), marital status (Married, Unmarried, and Other), drinking (No/Unknown, Yes), smoking (No/Unknown, Yes), diabetes (No/Unknown, Yes/Borderline), and hypertension (No/Unknown, Yes).

### 2.4 Statistical analysis

Demographic and clinical characteristics of participants were collected, with continuous variables described as (mean ± SD) and categorical variables expressed as number and percentage. Chi-squared tests were used to compare baseline characteristics of categorical variables. Logistic regression models were applied to investigate the relationship between CDAI and MetS in females. Model 1 was a rough model without no adjustment for any covariates. Model 2 was further adjusted for age, race and ethnicity, and education level. Model 3 was a fully adjusted model, further adjusting for alcohol consumption, smoking status, BMI, diabetes, and hypertension. To verify the robustness of the study results, a sensitivity analysis (Model 4) was conducted by excluding diabetes and hypertension to assess the impact of adjusting for these variables on the association between CDAI and MetS. A restricted cubic spline was then performed to explore the nonlinear relationship between the CDAI and MetS in females, and two-piecewise linear regression was used to calculate the inflection point. Finally, subgroup analysis was conducted to confirm the consistency and stability of the study results in each subgroup. All statistical analyses were conducted using R software version 4.2.3.^1^ Two-sided *p* < 0.05 was considered statistically significant.

## 3 Results

### 3.1 Baseline characteristics

In total, 2,790 female participants were included in this study and grouped according to the quartile distribution of CDAI. Significant trends were observed in race and ethnicity, education level, marital status, smoking status, and BMI with changes in CDAI quartiles. Additionally, the prevalence of MetS exhibited a significant downward trend with increased the CDAI quartiles (*p* < 0.001) (Table 1).

**Table 1:** Baseline analyses based on CDAI quartiles for general female adult population, NHANES 2011–2016.

*n*	Q1	Q2	Q3	Q4	*p*
	698	697	697	698	
**Age (y), mean (SD)**	49.90 (17.34)	49.15 (17.54)	49.05 (17.02)	48.73 (16.96)	0.632
**Race and ethnicity (%)**					0.015
Non-Hispanic Black	175 (25.1)	155 (22.2)	137 (19.7)	136 (19.5)	
Mexican American	77 (11.0)	98 (14.1)	97 (13.9)	87 (12.5)	
Other	170 (24.4)	157 (22.5)	162 (23.2)	206 (29.5)	
Non-Hispanic White	276 (39.5)	287 (41.2)	301 (43.2)	269 (38.5)	
**Education level (%)**					<0.001
Above high school	323 (46.3)	408 (58.5)	466 (66.9)	487 (69.8)	
Other	80 (11.5)	67 (9.6)	43 (6.2)	39 (5.6)	
High school	295 (42.3)	222 (31.9)	188 (27.0)	172 (24.6)	
**Marital status (%)**					0.003
Married	310 (44.4)	319 (45.8)	357 (51.2)	347 (49.7)	
Other	272 (39.0)	245 (35.2)	205 (29.4)	211 (30.2)	
Unmarried	116 (16.6)	133 (19.1)	135 (19.4)	140 (20.1)	
**Drinking (%)**					0.416
No/Unknown	305 (43.7)	294 (42.2)	274 (39.3)	292 (41.8)	
Yes	393 (56.3)	403 (57.8)	423 (60.7)	406 (58.2)	
**Smoking (%)**					<0.001
No/Unknown	401 (57.4)	465 (66.7)	477 (68.4)	485 (69.5)	
Yes	297 (42.6)	232 (33.3)	220 (31.6)	213 (30.5)	
**BMI (kg/m** ^**2**^ **), mean (SD)**	30.01 (7.49)	30.07 (7.63)	29.39 (7.55)	28.95 (7.64)	0.016
**Diabetes (%)**					0.228
No/Unknown	589 (84.4)	584 (83.8)	601 (86.2)	609 (87.2)	
Yes/Borderline	109 (15.6)	113 (16.2)	96 (13.8)	89 (12.8)	
**Hypertension (%)**					0.050
No/Unknown	417 (59.7)	431 (61.8)	464 (66.6)	448 (64.2)	
Yes	281 (40.3)	266 (38.2)	233 (33.4)	250 (35.8)	
**MetS (%)**					<0.001
No	427 (61.2)	431 (61.8)	463 (66.4)	502 (71.9)	
Yes	271 (38.8)	266 (38.2)	234 (33.6)	196 (28.1)	

### 3.2 Multivariate adjusted logistic regression

Multivariate logistic regression models were established to examine the relationship between the CDAI and MetS. Three models were constructed. Model 1 was not adjusted for any covariates. Model 2 was adjusted for age, race and ethnicity, and education level. Model 3 was further adjusted for alcohol consumption, smoking status, BMI, diabetes, and hypertension. In both Models 1 and 2, the CDAI was associated with a reduced prevalence of MetS, whether treated as a continuous or categorical variable. Even after adjusting for all confounding variables in Model 3, the protective effect of the CDAI (continuous) on MetS remained significant (odds ratio [OR] [95% confidence interval CI] = 0.96 [0.94, 0.99], *p* = 0.009). Compared with Q1, the CDAI in Q4 was associated with a 28% reduction in the prevalence of MetS (*p* = 0.017; Table 2). In the sensitivity analysis (Model 4, excluding diabetes and hypertension), the negative association between CDAI and MetS remained statistically significant (OR [95% CI] = 0.97 [0.94, 0.99], *p* = 0.009). The effect estimates for CDAI quartiles were comparable to those in Model 3, indicating that the association between CDAI and MetS remains robust regardless of whether diabetes and hypertension are adjusted for. In sum, the higher CDAI was a protective factor in MetS.

**Table 2:** Multivariable-adjusted logistic regression analysis of the relationship between CDAI and prevalence of MetS in American adult women, NHANES 2011–2016.

	Model 1		Model 2		Model 3		Model 4 (Sensitivity Analysis)	
	OR [95% CI]	*p*	OR [95% CI]	*p*	OR [95% CI]	*p*	OR [95% CI]	*p*
Q1	Ref	–	Ref	–	Ref	–	Ref	–
Q2	0.97 [0.78, 1.21]	0.800	1.05 [0.83, 1.33]	0.652	1.06 [0.82, 1.38]	0.645	1.08 [0.84, 1.39]	0.533
Q3	0.80 [0.64, 0.99]	0.041	0.90 [0.71, 1.14]	0.378	0.97 [0.75, 1.26]	0.831	0.97 [0.75, 1.25]	0.784
Q4	0.62 [0.49, 0.77]	<0.001	0.70 [0.55, 0.98]	0.004	0.72 [0.54, 0.94]	0.017	0.73 [0.56, 0.95]	0.017
CDAI	0.95 [0.93, 0.97]	<0.001	0.96 [0.94, 0.98]	0.001	0.96 [0.94, 0.99]	0.009	0.97 [0.94, 0.99]	0.009

Model 1 was not adjusted.

Model 2 was adjusted for age, race and ethnicity, and education.

Model 3 was adjusted for age, race and ethnicity, education, marital status, smoking, drinking, diabetes, and hypertension.

Model 4 (Sensitivity Analysis): Adjusted for Age, Race, Education, Marital Status, Smoking, and Drinking (excluding Diabetes and Hypertension).

The relationship between components of the CDAI and MetS was also examined. After adjusting for all confounders, no components (vitamins A, C, E, selenium, zinc, and carotenoids: α-carotene, β-carotene, lycopene, lutein, and zeaxanthin) were independently associated with the presence of MetS (*p* > 0.05; Table 3).

**Table 3:** Relationship between individual dietary antioxidants and prevalence of MetS in American adult women, NHANES 2011–2016.

	Model 1		Model 2		Model 3	
	OR [95% CI]	*p*	OR [95% CI]	*p*	OR [95% CI]	*p*
Vitamin A	1.00 [1.00, 1.00]	0.753	1.00 [1.00, 1.00]	0.646	1.00 [1.00, 1.00]	0.163
Vitamin C	1.00 [1.00, 1.00]	0.687	1.00 [1.00, 1.01]	0.408	1.00 [1.00, 1.00]	0.714
Vitamin E	0.99 [0.97, 1.01]	0.220	0.99 [0.97, 1.01]	0.308	0.98 [0.96, 1.01]	0.117
Selenium	1.00 [1.00, 1.00]	0.819	1.00 [1.00, 1.00]	0.079	1.00 [1.00, 1.00]	0.522
Zinc	0.99 [0.97, 1.01]	0.343	0.99 [0.97, 1.01]	0.467	0.99 [0.96, 1.01]	0.186
Alpha-carotene	1.00 [1.00, 1.00]	0.493	1.00 [1.00, 1.00]	0.868	1.00 [1.00, 1.00]	0.397
Beta-carotene	1.00 [1.00, 1.00]	0.135	1.00 [1.00, 1.00]	0.063	1.00 [1.00, 1.00]	0.236
Lycopene	1.00 [1.00, 1.00]	0.195	1.00 [1.00, 1.00]	0.116	1.00 [1.00, 1.00]	0.141
Lutein and zeaxanthin	1.00 [1.00, 1.00]	0.118	1.00 [1.00, 1.00]	0.170	1.00 [1.00, 1.00]	0.302

Model 1 was not adjusted.

Model 2 was adjusted for age, race and ethnicity, and education.

Model 3 was adjusted for age, race and ethnicity, education, marital status, smoking, drinking, diabetes, and hypertension.

### 3.3 Nonlinear relationship

**Figure 2:**
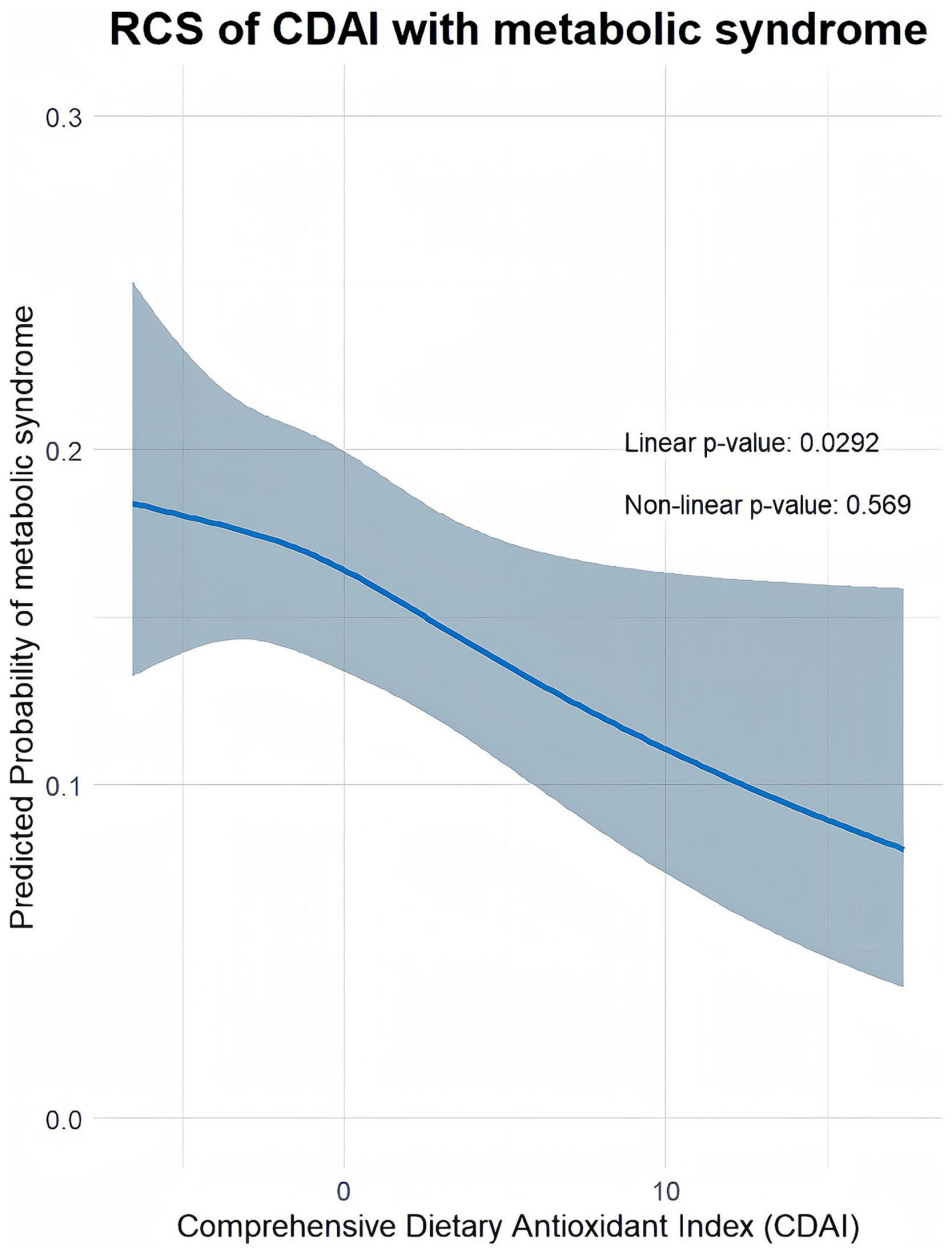
Exploration of nonlinear associations between CDAI and MetS.

A restricted cubic spline was used to analyze whether there was a nonlinear correlation between the CDAI and MetS. After adjusting for age, race and ethnicity, education level, alcohol consumption, smoking status, BMI, diabetes, and hypertension, the results showed that the relationship between the CDAI and MetS was linear (*p* for linearity = 0.0292, *p* nonlinearity = 0.569) (Figure 2).

**Figure 3:**
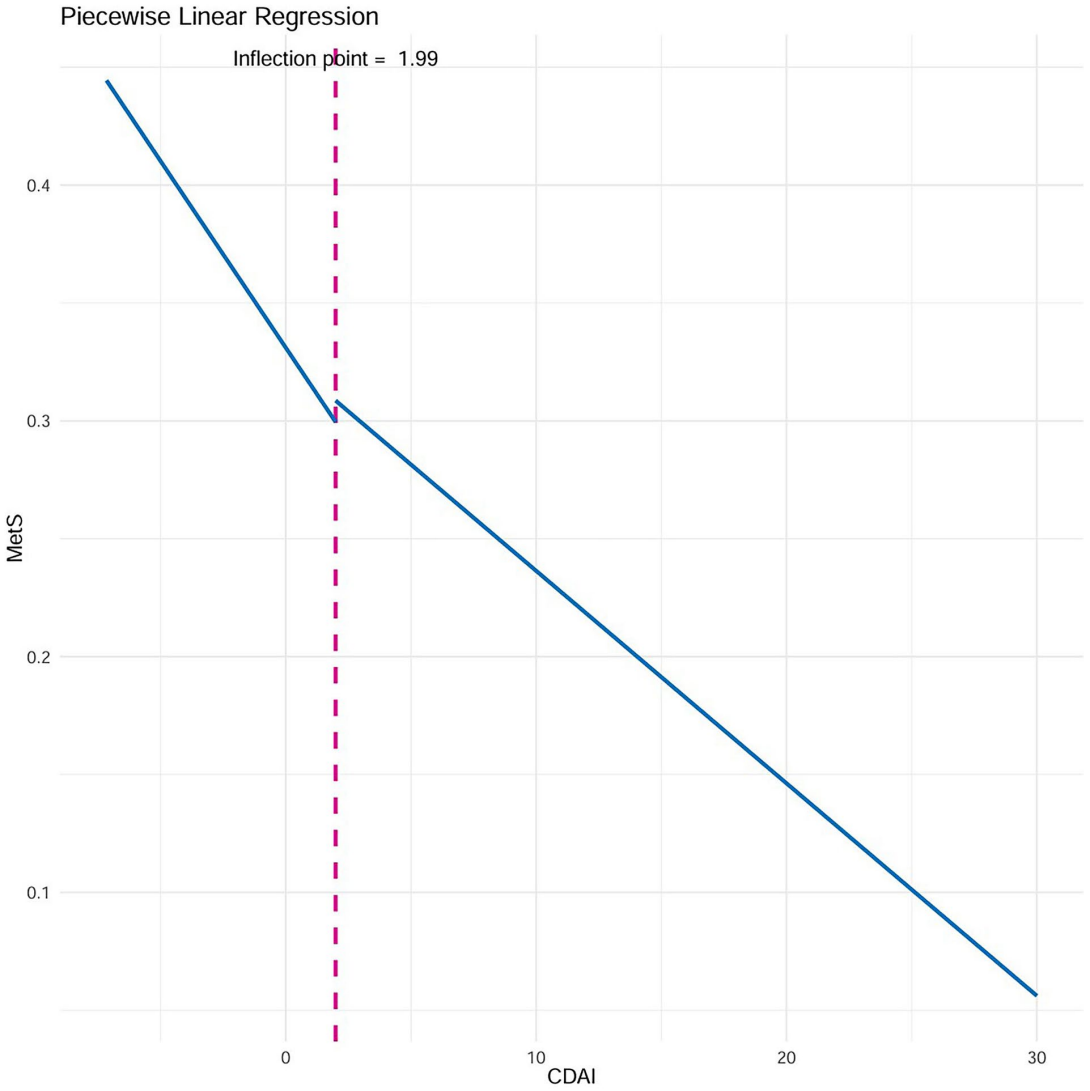
Two-piecewise linear regression.

The threshold effect of the CDAI on MetS in females was further analyzed using two-piecewise linear regression. The results showed that the inflection point of two-piecewise linear regression was 1.99, with a significant correlation between the CDAI and MetS in females (*p* < 0.05). When the CDAI was below 1.99, the risk of MetS in females decreased by 2% for each unit increase; with CDAI scores above 1.99, the risk reduction was 1% (Figure 3 and Table 4).

**Table 4:** Threshold effect analysis of CDAI on MetS using two-piecewise linear regression.

Inflection point	Adjusted OR (95% CI)	*p*-value
≤1.99	0.98 (0.98, 0.99)	<0.001
>1.99	0.99 (0.99, 1.00)	0.001
Log-likelihood ratio	0.001	

**Figure 4:**
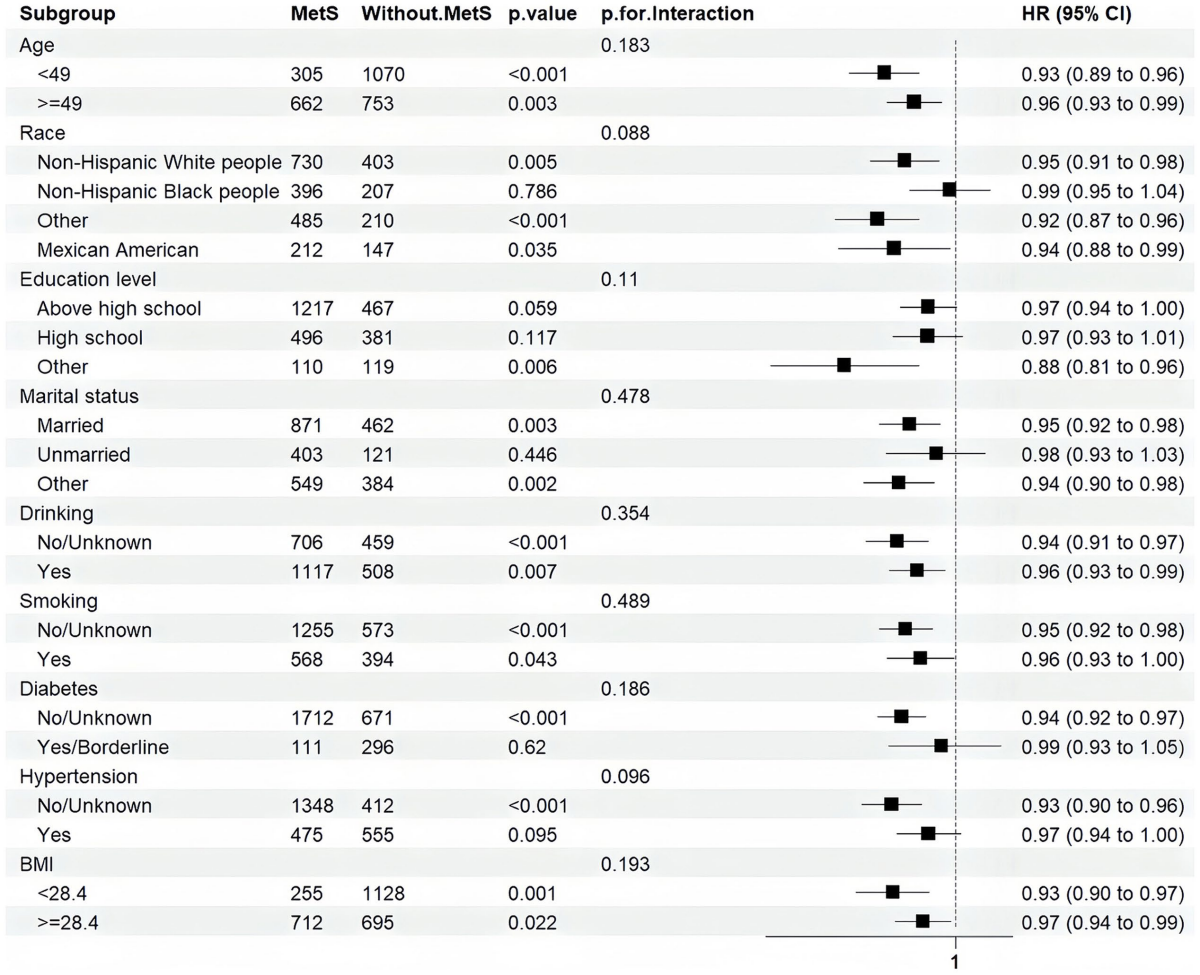
Subgroup analysis.

The results of subgroup analysis showed that the relationship between the CDAI and MetS was consistent and stable across all subgroups (Figure 4). None of the factors significantly affected the association (*p* > 0.05). Moreover, this association was found in all subgroups except for non-Hispanic Black people, participants with more than a high school education, unmarried participants, those with borderline or diagnosed diabetes, and participants with hypertension.

### 3.4 Relationship between estrogen and MetS

**Figure 5:**
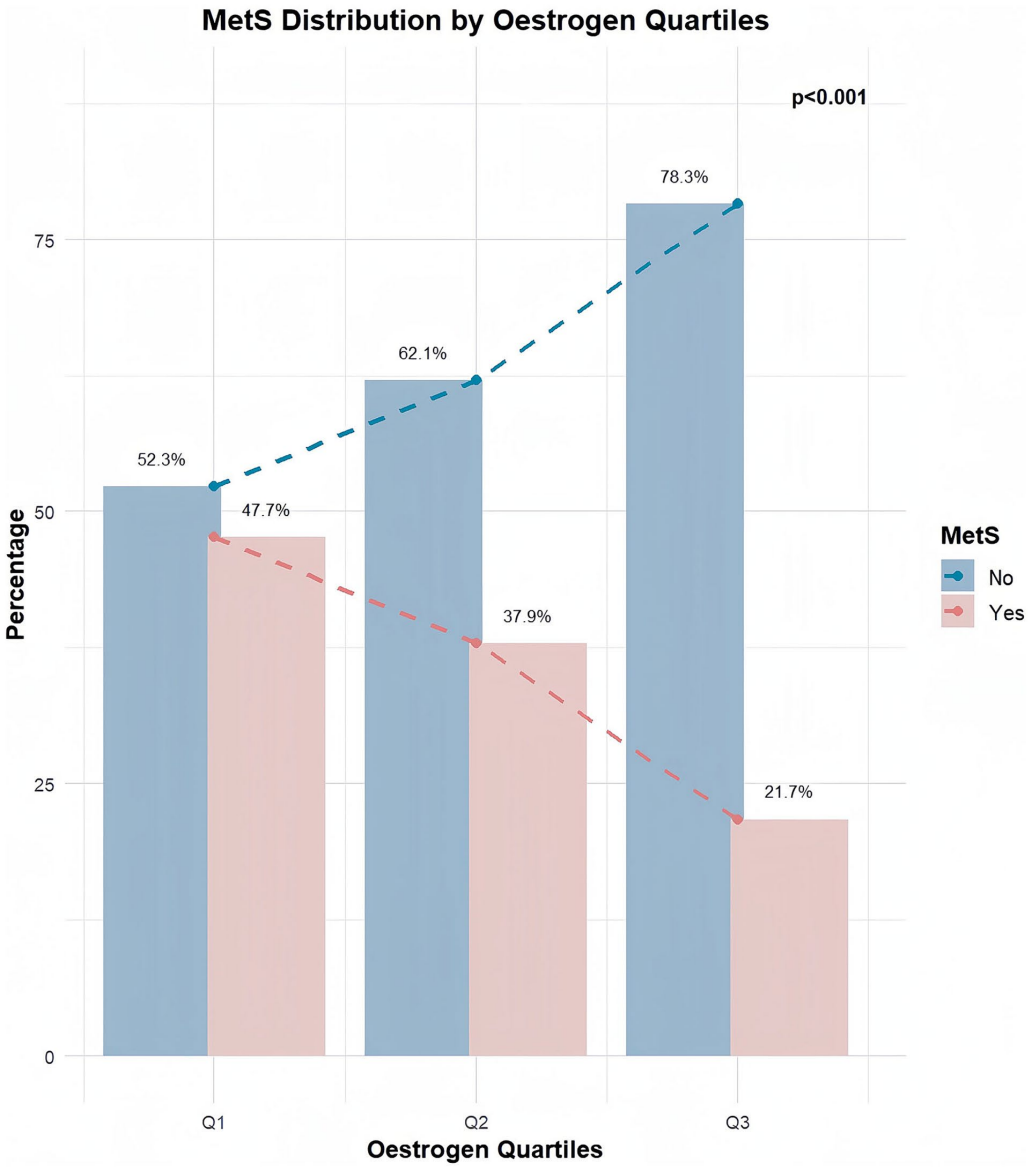
Relationship between estrogen and MetS.

In total, 1,562 participants (mean age: 47.23 years) with data for estrogen levels were screened from the 2,790 included participants. We explored whether there was a relationship between estrogen and MetS. In total, 559 participants were diagnosed with MetS, resulting in a prevalence rate of 35.79% (Table 5). The results indicated that females without MetS were younger (43.49 ± 16.04 years) and had lower BMI (28.29 ± 7.23 kg/m^2^) than those who had MetS (age: 54.77 ± 15.52 years, BMI: 34.37 ± 7.57 kg/m^2^). Additionally, females without MetS had higher CDAI scores. When estrogen was divided into tertiles, there was a significant downward trend in the prevalence of MetS with increasing estrogen tertiles (*p* < 0.001; Figure 5).

**Table 5:** Demographic and clinical characteristics of general female adult population (with estrogen) in the United States.

	Without MetS	MetS	*p*
*n*	1,003	559	
**Age (y), mean (SD)**	43.49 (16.04)	54.77 (15.52)	<0.001
**Race and ethnicity (%)**			0.017
Non-Hispanic Black	217 (21.6)	110 (19.7)	
Mexican American	143 (14.3)	103 (18.4)	
Other	254 (25.3)	111 (19.9)	
Non-Hispanic White	389 (38.8)	235 (42.0)	
**Education level (%)**			<0.001
Above high school	662 (66.0)	272 (48.7)	
Other	56 (5.6)	69 (12.3)	
High school	285 (28.4)	218 (39.0)	
**Marital status (%)**			<0.001
Married	492 (49.1)	280 (50.1)	
Other	280 (27.9)	216 (38.6)	
Unmarried	231 (23.0)	63 (11.3)	
**Drinking (%)**			<0.001
No/Unknown	378 (37.7)	273 (48.8)	
Yes	625 (62.3)	286 (51.2)	
**Smoking (%)**			0.001
No/Unknown	684 (68.2)	333 (59.6)	
Yes	319 (31.8)	226 (40.4)	
**BMI (kg/m** ^**2**^ **), mean (SD)**	28.29 (7.23)	34.37 (7.57)	<0.001
**Diabetes (%)**			<0.001
No/Unknown	942 (93.9)	406 (72.6)	
Yes/Borderline	61 (6.1)	153 (27.4)	
**Hypertension (%)**			<0.001
No/Unknown	754 (75.2)	262 (46.9)	
Yes	249 (24.8)	297 (53.1)	
**CDAI, mean (SD)**	−0.39 (3.87)	−1.10 (3.25)	<0.001
**Estrogen (pg/mL), mean (SD)**			<0.001
Q1	273 (27.2)	249 (44.6)	
Q2	323 (32.2)	197 (35.2)	
Q3	407 (40.6)	113 (20.2)	

**Figure 6:**
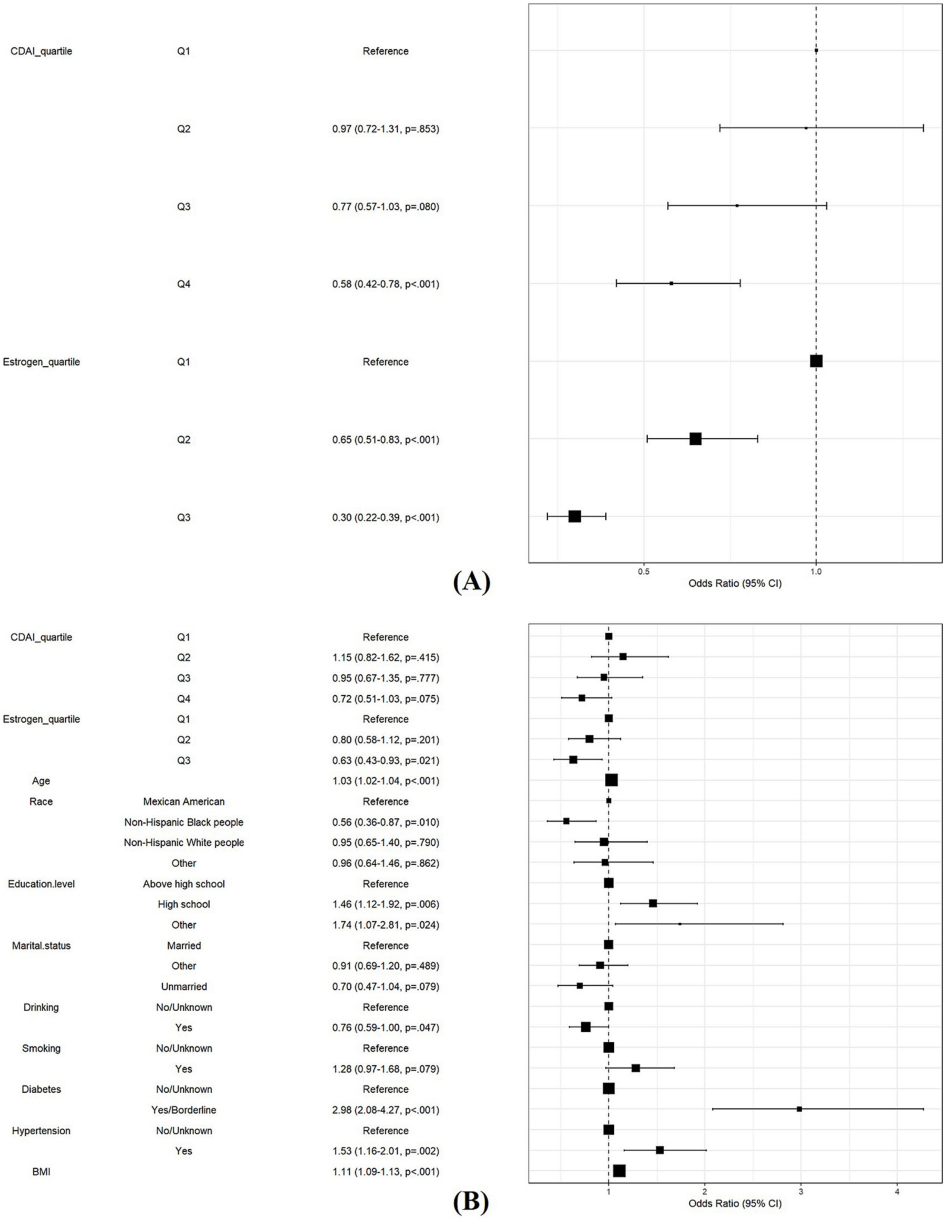
Role of estrogen in the association between CDAI and risk of MetS in women. **(A)** Model 1: Logistic regression analysis of CDAI and estrogen. **(B)** Model 2: Model 1 with additional covariates, including age, race and ethnicity, education, marital status, smoking, drinking, diabetes, and hypertension.

To investigate the role of estrogen in the association between CDAI and the risk of MetS in females, we performed further logistic regression analyses. Model 1 showed that both high levels of CDAI and high levels of estrogen were protective against MetS (OR [95% CI] 0.58 [0.42, 0.78], *p* < 0.001; OR [95% CI] = 0.30 [0.22, 0.38], *p* < 0.001, respectively) (Figure 6A). After adjusting for all covariates in Model 2, the protective effect of estrogen remained significant (OR [95% CI] 0.63 [0.43, 0.93], *p* = 0.021) (Figure 6B).

## 4 Discussion

Global epidemiological data reveal a striking female preponderance in MetS, affecting over 500 million females worldwide (4, 5, 37). This disparity may stem from estrogen’s role in redox regulation, as estrogen deficiency elevates oxidative stress and inflammation—canonical drivers of metabolic dysregulation (38–40). More critically, estrogen deficiency disrupts the estrogen-antioxidant crosstalk, a novel mechanism identified in this study. Our investigation elucidates a critical interplay between CDAI and estrogenic status in modulating MetS risk among females. We identified a robust inverse association between CDAI and MetS prevalence, characterized by a biphasic dose–response relationship with an inflection point at CDAI = 1.99. Crucially, estrogen status significantly modified this relationship, with higher estrogen levels enhancing the protective capacity of CDAI. These findings underscore the necessity of optimizing antioxidant intake during premenopausal phase to maximize metabolic protection.

MetS is characterized by a heightened pro-oxidative and pro-inflammation (41). Oxidative stress arises owing to an imbalance between the synthesis of antioxidants and pro-oxidants, causing harm to tissues and organs. It primarily results from excessive levels of reactive oxygen species (ROS), which cause damage to damage macromolecules such as DNA, lipids, proteins, and carbohydrates (42, 43). As an extrinsic factor, diet can affect the plasma redox status by reducing ROS and reactive nitrogen species (44). The CDAI serves as an integrative measure of dietary antioxidant vitamins/minerals, indicating the antioxidant potential of individual dietary sources. We analyzed both the individual components of the CDAI and the index as a whole. In separate analyses, no single dietary component was significantly associated with MetS prevalence (*p* > 0.05), a finding that contrasts with previous studies (45). This may be due to the differences in the cohort samples of our study, which was conducted on females only, and previous population-wide studies. However, when considering the CDAI as a whole, we observed a significant downward trend in MetS prevalence across increasing CDAI quartiles (*p* < 0.001). The risk of MetS in females decreased significantly with higher CDAI levels, and females in the highest quartile (OR [95% CI] = 0.62[0.49, 0.77], *p* < 0.001) of the CDAI had an approximately 28% lower risk of MetS than those in the lowest quartile. This is consistent with previous population-wide studies, and such an association was also present in female patients (35, 36, 44). Our analyses revealed a nonlinear association between the CDAI and MetS, with an inflection point identified at 1.99 through threshold effect modeling. Below this critical threshold, each unit increase in CDAI conferred a 2.0% absolute risk reduction (*p* < 0.001), whereas supra-threshold increments attenuated this protective to 1.0% per unit (*p* = 0.001), suggesting a biological ceiling effect of dietary antioxidants. In previous studies, a dose–response trend similar to that in our study was observed (36). Therefore, we hypothesized that a high intake of dietary antioxidants and phytochemicals may reduce the risk of developing MetS in females because the combined intake of dietary antioxidants reduces oxidative stress. Intake of exogenous antioxidants can improve patient quality of life by preventing oxidative imbalance and maintaining a stable biochemical redox state (46), thereby avoiding the deleterious effects of chronic oxidative stress in the human body (47). According to a previous study, daily intake of antioxidants can enhance antioxidant defense and mitigate oxidative stress by increasing plasma antioxidant levels (48).

Oxidative stress exhibits sex-related differences, with estrogen conferring protection against its detrimental effects in females (49, 50). This hormonal regulation plays a pivotal role in modulating the protective association between dietary antioxidants (assessed via CDAI) and MetS in females. Estrogen regulates redox homeostasis through multiple molecular mechanisms, including: upregulating the expression of endogenous antioxidant enzymes (51), binding to mitochondrial estrogen receptors to enhance antioxidant defense (52), and suppressing reactive oxygen species (ROS) generation by inhibiting NADPH oxidase activity (52). Epidemiological studies indicate that the prevalence of MetS increases with age (53), a phenomenon largely driven by age-related exacerbation of oxidative stress driven by either excessive ROS production or impaired antioxidant systems (54).

In our study, the mean age of females without MetS was 43.49 ± 16.04 years, significantly lower than that of females with MetS (54.77 ± 15.52 years; *p* < 0.001). This age disparity aligns with epidemiological evidence showing a higher prevalence of MetS in postmenopausal populations (53, 55). Mechanistically, menopause typically occurs between 45 and 55 years (55), and the subsequent decline in estrogen levels exacerbates oxidative stress and systemic inflammation (38, 39). Estrogen deficiency disrupts redox homeostasis, leading to progressive accumulation of oxidative damage markers (56, 57), which in turn promotes endothelial dysfunction—a key contributor of insulin resistance, hypertension, and dyslipidemia (45, 57). These pathological alterations collectively drive MetS development in aging females.

Meanwhile, our study confirmed this finding, demonstrating a significant downward trend in MetS prevalence with increasing estrogen levels. Regression analysis further revealed that high levels of estrogen exerted a protective effect against MetS (OR[95% CI] = 0.30[0.22, 0.38], *p* < 0.001). This protective role may be attributed to the loss of estrogen is associated with a diminished defense against oxidative stress (58). The oxidative stress has a pivotal role in some components of MetS, including abdominal obesity, hypertension, insulin resistance, and dyslipidemia (44, 59–61).

This was the first large-sample study to investigate the association between the CDAI and MetS in females, while also considered the role of estrogen. Our findings provide epidemiological evidence supporting the implementation of preemptive nutritional strategies aimed at optimizing composite dietary antioxidant optimization during the estrogen depletion phase (specifically the premenopausal transition window) as a prophylactic measure against MetS development. A public health priority for the prevention of disease is consuming an optimal diet that can reduce or suppress inflammation owing to its composition and thereby modulate the risk of various diseases (62). As a new paradigm in the prevention and treatment of MetS, dietary interventions should include advice on antioxidant-rich diets given by nutritional professionals as well as increased promotion of these diets and specific antioxidant dietary modifications for menopausal females. Increased dietary intake of vitamins A, C, and E together with selenium, zinc, and carotenoid-rich foods may improve the current status of MetS among females globally.

This study benefits from methodological rigor through utilization of the nationally representative NHANES sampling framework and comprehensive adjustments for established demographic, anthropometric, and metabolic covariates, enhancing internal validity. Notwithstanding these strengths, several methodological constraints warrant consideration: (1) The observational cross-sectional design precludes temporal sequence determination and causal inference; (2) Sex-specific analytic focus limits external validity to male populations; (3) The moderate sample size relative to population-level epidemiological standards constrains statistical power for detecting modest effect sizes; (4) Potential residual confounding persists despite multivariable adjustments, including unmeasured lifestyle determinants (e.g., chrononutrition patterns, physical activity gradients) and epigenetic regulatory mechanisms influencing redox homeostasis.

## Data Availability

The datasets presented in this study can be found in online repositories. The names of the repository/repositories and accession number(s) can be found in the article/supplementary material.

## Footnotes

^1^ https://www.R-project.org
